# Reproducibility analysis on shear wave elastography (SWE)-based quantitative assessment for skin elasticity

**DOI:** 10.1097/MD.0000000000006902

**Published:** 2017-05-12

**Authors:** Yang Sun, Chuan Ma, XiaoLong Liang, Run Wang, Ying Fu, ShuMin Wang, LiGang Cui, ChunLei Zhang

**Affiliations:** a Department of Ultrasound; b Department of Dermatology, Peking University Third Hospital, Beijing, P.R. China.

**Keywords:** elastography, reproducibility, skin, subcutaneous tissue, ultrasound

## Abstract

Shear Wave Elastography (SWE) is an objective and non-invasive method widely used to quantify the tissue solidity. However, there are concerns about the accuracy of the skin SWE results due to the low signal-to-noise ratio (SNR) caused by subcutaneous fat, muscle and bone. This article analyzed the reproducibility of the result for skin SWE and therefore evaluated the availability of SME for skin elasticity involved diseases. Thirty volunteers (mean age: 37 ± 12 years) were selected. SWE were taken on the skin of abdomen and the middle tibia in order to assess the impact of fat, muscle and bone on SWE results. Skin in the area of anterior and lateral tibia marked with seven parallel lines, and each line indicated an identical thickness of the subcutaneous fat from 1–7 mm. Intra-class correlation coefficients (ICC) were used to evaluate the intra-observer and inter-observer reproducibility. The solidity of abdominal skin showed soft and small individual differences (12.4 ± 2.7 kPa), whereas high shear moduli (25–48 kPa) were observed in the skin above tibia and tibialis anterior muscle. When the subcutaneous fat was thicker than 3 mm (≥3), we obtained excellent intra-observer reproducibility (ICC range 0.78–0.98) and inter-observer reproducibility (ICC range 0.75–0.98). The thickness of subcutaneous fat could affect the reproducibility of skin SWE. The further study on skin SWE standardization should be taken.

## 1 Introduction

Skin elasticity is correlated with aging and trauma.^[1,2]^ Under pathologic conditions, changes in the fatty and fibrous tissue components of skin lead to a change in its elastic moduli. Quantitative assessment of skin elasticity could show skin lesion status. For example, solidity of skin lesion various in different stage of systemic scleroderma and wound healing. Therefore, solidity can be used to evaluate the therapeutic efficacy.^[3,4]^ Over the past 3 decades, indentometry has been widely used to measure tissue solidity.^[2]^ However, it is a subjective test which has low accuracy and reproducibility, and limited in special area of skin.^[5]^ Cutometer is a reliable tool for skin elastic detection, but it can only measure a small area of scar, is not representative.^[6]^

Shear wave elastography (SWE) is an objective and noninvasive method used to quantify the tissue solidity. Compared with indentometry, SWE is applicable to the skin of whole body.^[7]^ Previous studies show wide application of SWE in liver fibrosis,^[8]^ breast lesions,^[9]^ prostate cancers,^[10]^ and muscle property.^[11]^ Recently, some preliminary study reported SWE can be used to diagnosis benign or malignant solid skin tumors.^[12]^

However, there are arguments about the reproducibility and standardization of SWE for skin detection. In particular, when SWE is used to evaluate cutaneous lesions, the probe and the skin is too close^[13]^ that some subcutaneous tissues may cause stronger signal to override the signal of skin detection, and ultimately results in a low signal-to-noise ratio. How subcutaneous fat, muscle, and bones influence the measurement is still unknown, whereas our study is to verify the impact of these tissues on skin SWE reproducibility by assessment of intra- and interobserver agreement.

## 2 Materials and methods

### 2.1 Volunteers

We performed a prospective analysis of 30 volunteers who were involved in this study with their consents. The inclusion criteria of the volunteers were listed as follows: age range of 20 to 70 years, agreement of a 1-hour elastography scan, no previous skin diseases or trauma, and the thickness of subcutaneous fat above the tibia was no more than 1 mm. This study was approved by the relevant local ethics committee.

### 2.2 SWE measurement

All imaging procedures were performed with an ultrasound system (SuperSonicImagine, Aix en Provence, France) by a Superlinear^TMSL^15-4 transducer. The experiments incorporated the default SWE settings, with the tissue shear modulus values reported in kilopascals (kPa). The shear modulus scale was adjusted to enrich the color of the region of interest, with soft tissues indicated by dark blue, and areas of increasing stiffness indicated in the following order: light blue, green, orange, and red.

### 2.3 Protocols

Participants were scanned by 2 sonologists: one with 5 years’ experience and the other with no SWE experience. Sonologists were asked to scan a given location using the minimized transducer pressure. A large amount of gel was applied between the probe and the skin to limit tissue deformation induced by the operators. During scanning, the 2 sonologists were asked to record a 10-second video after experiencing an SWE cine loop static period of ≥10 seconds, for the visualization of the homogeneous elastic modulus color signal in each layer. Another sonologist, who was blinded to the results of the previous attempts, selected a satisfactory cine loop and traced the margin of the chosen location. Subsequently, the integrated SWE software automatically determined the shear modulus data for the traced Q-box, including the maximum, minimum, mean, and standard deviation values. The mean values of skin shear moduli in the scanned region were recorded. All of procedures were repeated again on the next day.

**Figure 1:**
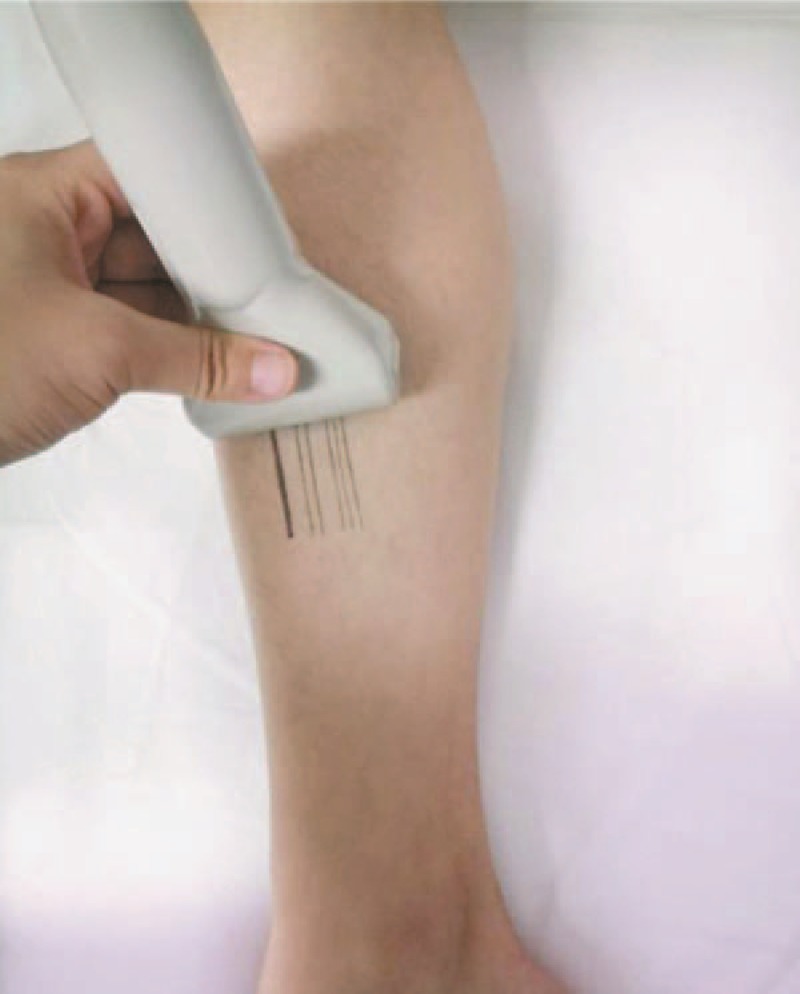
The method used to mark the 7 parallel lines at 1 mm intervals with its corresponding gray scan image (Fig. 2). Measurement of the elastic modulus in the areas with different thickness of subcutaneous fat followed by marked lines.

**Figure 2:**
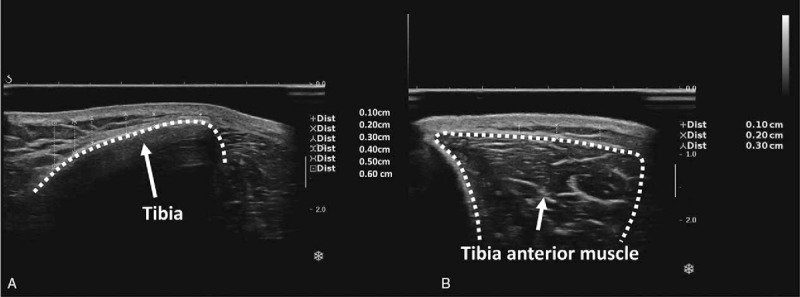
(A) and (B) are the sonograms when assessing the skin above anterior and lateral tibia. The thickness of subcutaneous fat above tibia and tibialis anterior muscle showed a graded increasing trend.

Three skin areas included abdomen, anterior, and lateral tibia (in the middle area) in order to clarify the influence of different tissue (fat, muscle, and bone, respectively) on SWE measurement. The ultrasound transducer was placed at the umbilicus level perpendicular to the long body axes. The skin was marked with 7 parallel lines to indicate the thickness of the subcutaneous fat ranging from 1 to 7 mm in the anterior and lateral tibia (Figs. 1 and 2).

### 2.4 Statistical analysis

The SPSS software (version 20.1, SPSS, IBM) was used for data analysis. Continuous variables were expressed as mean ± standard deviation. Intraclass correlation coefficients (ICCs) were used to evaluate the intra- and interobserver reproducibility. ICC values of <0.40 indicated poor agreement; 0.40 to 0.75 fair to good agreement; and >0.75 excellent agreement.

## 3 Result

### 3.1 Population

Thirty volunteers (15 females and 15 males) were enrolled in this study. The mean age of the volunteers was 37 ± 12 years.

### 3.2 Quantitative analysis

#### 3.2.1 Elastography

In volunteers, the mean skin shear modulus values ranged from 12.4 to 50.2 kPa. Table 1 shows the overall average elastic moduli for each skin area. The solidity of skin in the abdomen group displayed soft and small individual differences (12.4 ± 2.7 kPa), whereas high shear moduli (25–48 kPa) were observed in the anterior and lateral groups. Furthermore, the thinner the subcutaneous fat was, the higher the shear moduli and standard deviations were obtained.

**Table 1:**
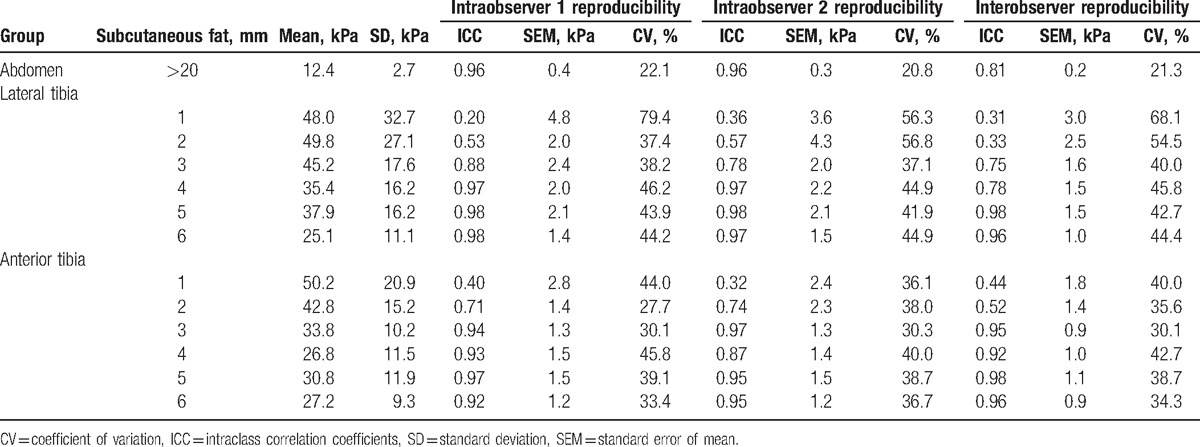
Shear wave elastography values and reproducibility in different skin area.

#### 3.2.2 Within-observer reproducibility

The intraobserver reproducibility of skin SWE on abdomen was considered excellent (ICC = 0.96). When the subcutaneous fat was thicker than 3 mm, we obtained excellent agreement of intraobserver reproducibility (ICC range 0.78–0.98). The intraoperator reproducibility was poor to good agreement (ICC range 0.20–0.74) when the thickness of subcutaneous fat not more than 2 mm.

#### 3.2.3 Interoperator reproducibility

The overall interobserver agreement was lower than the intraobserver reproducibility. However, the abdomen group showed an excellent ICC of 0.81. In the anterior and lateral tibia group, poor agreement (ICC range 0.31–0.52) was observed when the thickness of subcutaneous fat was not more than 2 mm, excellent agreement was obtained in the areas of fat thicker than 3 mm (ICC range 0.75–0.98).

## 4 Discussion

SWE is a new technique to objective quantitative tissue solidity, which shows high reproducibility in liver, breast, prostate, muscle, and so on. All of these studies confirm that excellent observation agreements come from standardized assessing, such as choosing the target areas with high signal-to-noise ratio, recognizing, and avoiding SWE artifacts. Theoretically, skin is an ideal material for SWE, based on the fact that soft tissue would gain higher accuracy than hard tissue.^[13]^ However, in different areas of the skin, subcutaneous tissues vary a lot and therefore have different impacts to the SWE results. Since the combined impacts to SWE by these tissues are still unclear, in this study we tried to explore the reproducibility of skin SWE measurements with fat, muscle, and bone separately.

Subcutaneous fat is relative isotropy and homogeneous which cause steady appearance of SWE. In the history of elastography, many studies regard subcutaneous fat as a control with malignant or benign nodular.^[14]^ In this study, the skin SWE of abdomen covered fatty layer (>20 mm) gained excellent agreement (0.81–0.96) in intra- and interobserver, confirmed that subcutaneous fat has little impact on skin SWE reproducibility. In physical structure, the thickness of subcutaneous fat above the anterior and lateral tibia shows a graded increasing trend from 1 to 6 mm (Fig. 2). Tibia and tibialis anterior muscle lies under this fat in transection, respectively. When the skin SWE of abdomen serves as a control with anterior and lateral tibia, the influence of tibia and tibialis anterior muscle to the skin SWE reproducibility can be explored.

The skin SWE reproducibility in anterior and lateral tibia area is lower than it in abdomen area confirmed that muscle and bone have higher influence to skin SWE than fat. In the areas of lower leg, if subcutaneous fat is thicker or equal to 3 mm, higher SWE reproducibility (excellent agreement) was observed; if the thickness of subcutaneous fat is less than 2 mm, the result is not stable (poor to good agreement observed). This result shows the influences by muscle and bone: since the elastic moduli of muscle and bone are apparently higher than skin within 1 to 2 mm spatial resolution,^[15]^ the signal of muscle and bone may override the signal of skin within the range of 1 to 2 mm. The lower skin SWE reproducibility may relate to the artifact of underlying muscle and bone. This hypothesis supported by previous study. Rosskopf et al^[11]^ indicated that the deep layer supraspinatus (proximal to the scapula) was observed low muscle SWE reproducibility. Thus, in this article we propose the minimal thickness of subcutaneous fat is 3 mm for the skin SWE. It could be a reference for the skin SWE measurement except for the area of tibia, such as skin in face, neck, and extremities. For example, the construction of face is so complex that would affect the skin SWE reproducibility. Appropriated choosing the area of skin SWE test would improve the credibility of skin solidity, so that diagnosis the skin aging and diseases.

It is widely accepted that skin solidity is related to genetic, age, sex, location, and environment condition.^[16]^ In our study, the SWE assessment of skin solidity showed higher values in the areas of anterior and lateral tibia than abdomen. The standard deviation values of skin elastic moduli are lower in abdomen than anterior and lateral tibia. This might be related to different skin tension. The skin tension is higher in lower leg than abdomen.^[17]^ Volunteers’ individual variations may also be responsible for the obvious standard deviations. In order to improve the comparability on skin SWE, an ideal testing area is required a low variety zone. For example, the skin SWE in abdomen could be a good control for skin aging or disease in the other area.

Because there is no way to control the tension of skin in vivo, the limitation of this study is fail to keep the same skin tension among different groups. The skin tension would impact SWE reproducibility. Therefore, the influence of muscle and bone would be overestimated. In this study, we failed to explore the reproducibility of skin SWE for obesity patient with bulged abdomen, owing to the small sample size. With the skin tension increased in bulged abdomen, the reproducibility of skin SWE in abdomen might be decreased. In addition, gold standard of quantitative skin solidity assessment in vivo is lack. The study was unable to validate the accuracy and sensitivity of the ultrasound elasticity measurements.

## 5 Conclusion

Skin SWE is an objective, noninvasive tool that would be convenient to observe the process of skin aging and diseases with solidity changing, such as photo-aging,^[18]^ scar,^[19]^ systemic sclerosis,^[20]^ lymphoma, and the other skin cancers.^[12]^ In this study, we obtained high reproducibility in tested areas when minimize the influence of muscle and bone. In addition, the further study on skin SWE standardization should be taken.
